# Supplementary material for: Automatic Phenotyping Using Exhaustive Projection Pursuit
Source: bioRxiv. 2025 Jun 6:2024.11.20.624581. Preprint. [Version 2] doi: 10.1101/2024.11.20.624581 (PMC12157688; doi:10.1101/2024.11.20.624581)
Supplement: Supplement 1 [file NIHPP2024.11.20.624581v2-supplement-1.pdf]

bioRxiv preprint doi: https://doi.org/10.1101/2024.11.20.624581; this version posted June 6, 2025. The copyright holder for this preprint (which was not certified by peer review) is the author/funder, who has granted bioRxiv a license to display the preprint in perpetuity. It is made available under aCC-BY 4.0 International license.

A Supplementary Figures

bioRxiv preprint doi: https://doi.org/10.1101/2024.11.20.624581; this version posted June 6, 2025. The copyright holder for this preprint (which was not certified by peer review) is the author/funder, who has granted bioRxiv a license to display the preprint in perpetuity. It is made available under aCC-BY 4.0 International license.

OMIP-077 Reference Gating

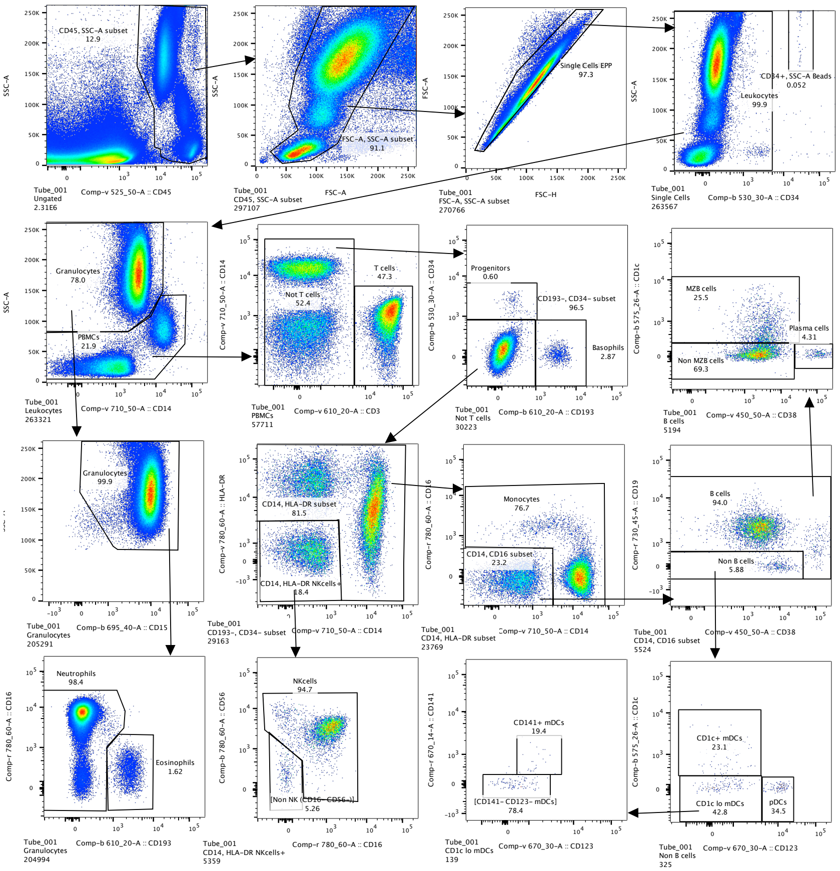

Figure 8: OMIP-077[5] Recreation of the Published Gate Sequence. Data transforms and gates were made to match those in Figure 1[5] of Boesch et al. The marker- dye-primary detector alignments for the OMIP-077 reagents are listed in Figure 9. (2.3 million events total)

bioRxiv preprint doi: https://doi.org/10.1101/2024.11.20.624581; this version posted June 6, 2025. The copyright holder for this preprint (which was not certified by peer review) is the author/funder, who has granted bioRxiv a license to display the preprint in perpetuity. It is made available under aCC-BY 4.0 International license.

OMIP-044 OMIP-077 Marker-Dye List

OMIP-044 Mair & Prlic

Marker Specificity	Fluorochrome	Detector
CCR7	PE	G575-A
CD1c	AF647	R660-A
CD3	BUV661	U660-A
CD4	PE-Cy7	G780-A
CD8	BB660	B660-A
CD11c	AF700	R710-A
CD14	BV711	V710-A
CD16	BUV496	U500-A
CD19	PE-Cy5.5	G710-A
CD45RA	BV570	V570-A
CD45	BUV805	U780-A
CD56	BUV563	U570-A
CD80	PE-Cy5	G660-A
CD123	BV786	V780-A
dead	UV Blue	U450-A
HLA-DR	APC-H7	R780-A

OMIP-077 Boesch, et al

Marker Specificity	Fluorochrome	Detector
CD1c	PE	b 575_26-A
CD3	BV605	v 610_20-A
CD14	BV711	v 710_50-A
CD15	PerCP-Cy5.5	b 695_40-A
CD16	APC-efluor780	r 780_60-A
CD19	APC-R700	r 730_45-A
CD34	FITC	b 530_30-A
CD38	BV421	v 450_50-A
CD45	BV480	v 525_50-A
CD56	PE-Cy7	b 780_60-A
CD123	BV650	v 670_30-A
CD141	APC	r 670_14-A
CD193	PE-CF594	b 610_20-A
HLA-DR	BV786	v 780_60-A

Figure 9: Marker-dye-detector list for reagents used in reference and EPP gating for OMIP-044 and OMIP-077

bioRxiv preprint doi: https://doi.org/10.1101/2024.11.20.624581; this version posted June 6, 2025. The copyright holder for this preprint (which was not certified by peer review) is the author/funder, who has granted bioRxiv a license to display the preprint in perpetuity. It is made available under aCC-BY 4.0 International license.

OMIP-044 Reference Gating

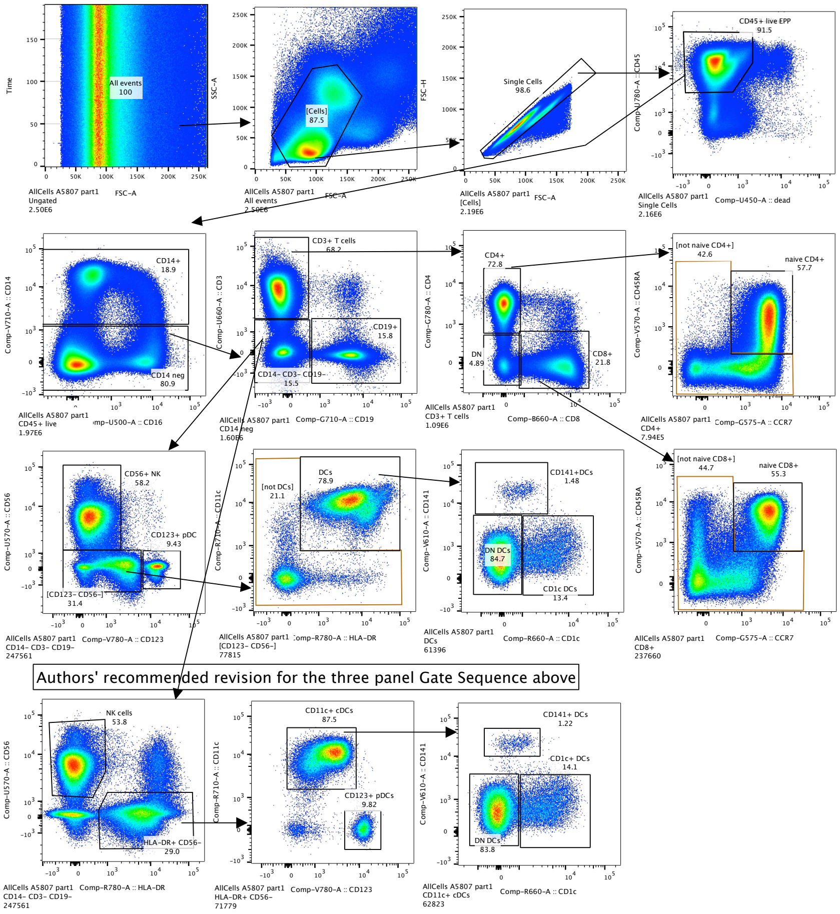

Figure 10: OMIP-044[3]. Recreation of the Published Gate Sequence. Data transforms and gates were made to match those in Figure 1[3] of Mair and Prlic. The three panels in the fourth row illustrate a revised gating strategy which provides better resolution of pDCs compared to the original three panels above them by reversing the order of HLADR and CD123 gating[14]. The marker-dye-primary detector alignments for the OMIP-044 reagents are listed in Figure 9. (2.5 million events total)

bioRxiv preprint doi: https://doi.org/10.1101/2024.11.20.624581; this version posted June 6, 2025. The copyright holder for this preprint (which was not certified by peer review) is the author/funder, who has granted bioRxiv a license to display the preprint in perpetuity. It is made available under aCC-BY 4.0 International license.

OMIP-044 EPP Gating-1

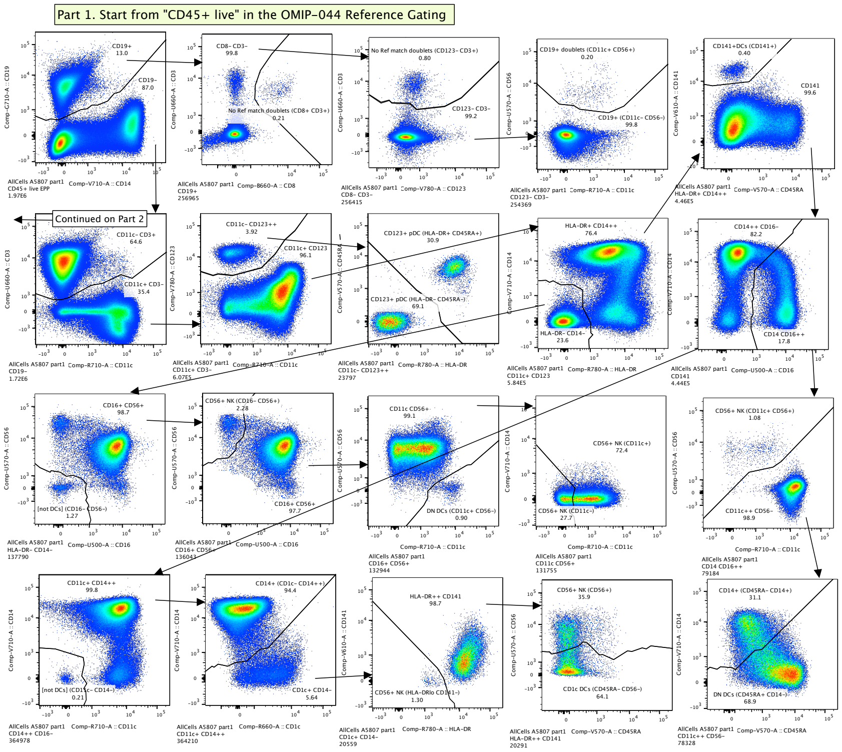

Figure 11: OMIP-044 EPP Gating Sequence - Part 1. EPP was started from the recreated reference gate “CD45+ live” shown in the upper right panel of Figure 8. The marker- dye-primary detector alignments for the OMIP-044 reagents are listed in Figure 9.

bioRxiv preprint doi: https://doi.org/10.1101/2024.11.20.624581; this version posted June 6, 2025. The copyright holder for this preprint (which was not certified by peer review) is the author/funder, who has granted bioRxiv a license to display the preprint in perpetuity. It is made available under aCC-BY 4.0 International license.

OMIP-044 EPP Gating-2

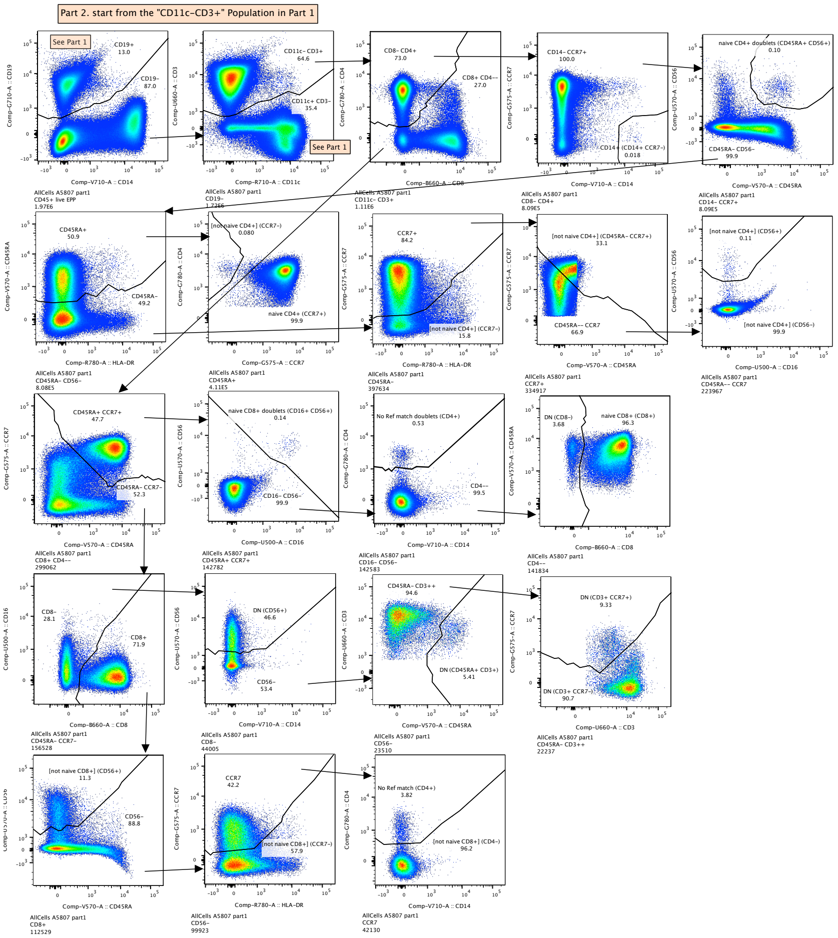

Figure 12: OMIP-044 EPP Gating Sequence - Part 2. This continues from the Part 1 population “CD11c- CD3+” (Figure 11, Row 2, Column 1).

bioRxiv preprint doi: https://doi.org/10.1101/2024.11.20.624581; this version posted June 6, 2025. The copyright holder for this preprint (which was not certified by peer review) is the author/funder, who has granted bioRxiv a license to display the preprint in perpetuity. It is made available under aCC-BY 4.0 International license.

OMIP-047 Reference Gating

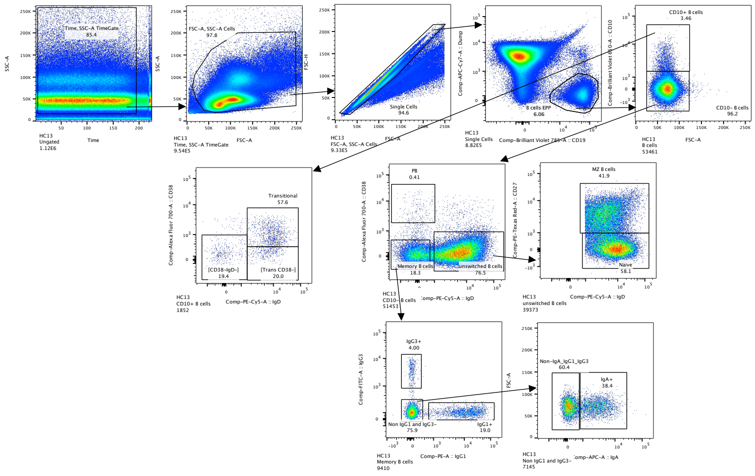

Figure 13: OMIP-047[4]. Recreation of the Published Gate Sequence. Data transforms and gates were made to match those in Figure 1[4] of Liechti et al. (112,000 events total)

bioRxiv preprint doi: https://doi.org/10.1101/2024.11.20.624581; this version posted June 6, 2025. The copyright holder for this preprint (which was not certified by peer review) is the author/funder, who has granted bioRxiv a license to display the preprint in perpetuity. It is made available under aCC-BY 4.0 International license.

OMIP-047 EPP Gating

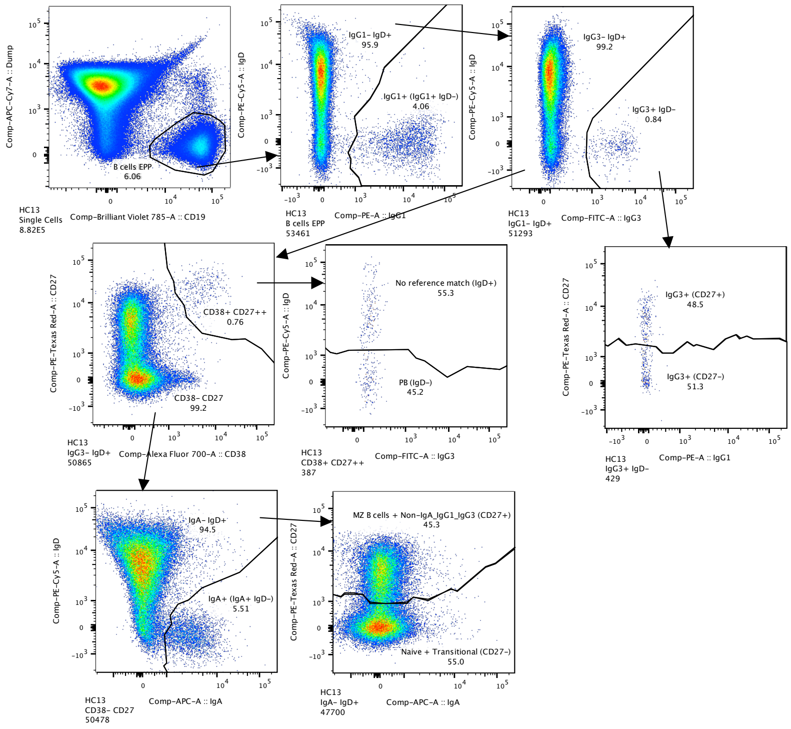

Figure 14: OMIP-047 EPP Gating Sequence. EPP was started from the recreated reference gate "B cells" as defined in the Row 1 Column 4 panel of Figure 13.

bioRxiv preprint doi: https://doi.org/10.1101/2024.11.20.624581; this version posted June 6, 2025. The copyright holder for this preprint (which was not certified by peer review) is the author/funder, who has granted bioRxiv a license to display the preprint in perpetuity. It is made available under aCC-BY 4.0 International license.

ESHGHI Reference Gating

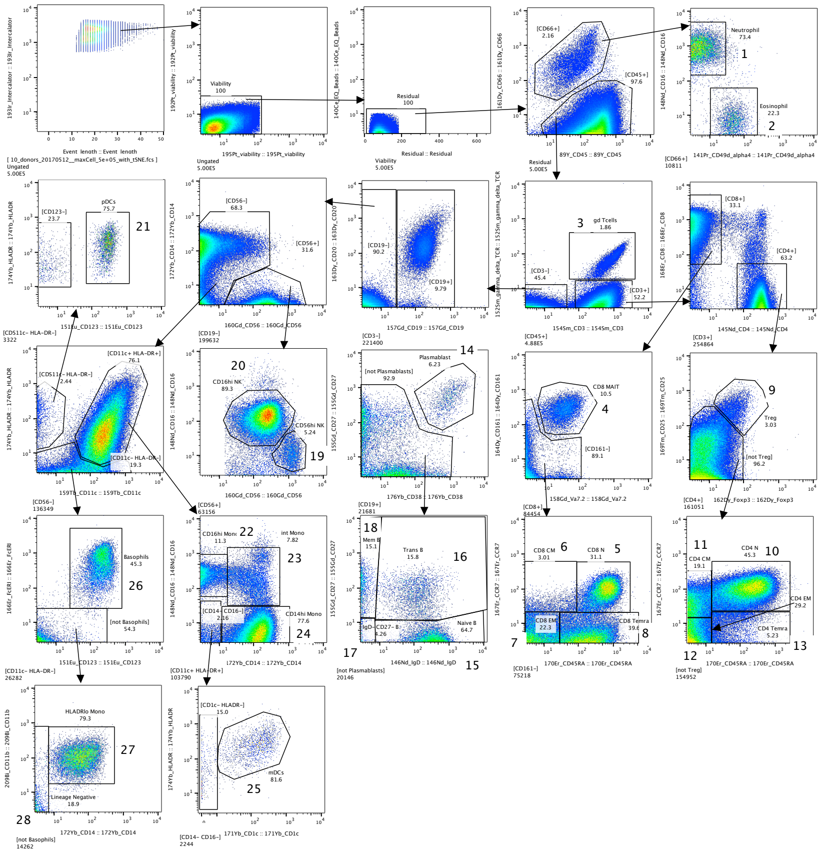

Figure 15: Eshghi, et al[6]. Recreation of the published gate sequence in Figure 1[6] of Eshghi, et al. The numbers and their associated population names match those defined in the reference figure. (500,000 events total)

bioRxiv preprint doi: https://doi.org/10.1101/2024.11.20.624581; this version posted June 6, 2025. The copyright holder for this preprint (which was not certified by peer review) is the author/funder, who has granted bioRxiv a license to display the preprint in perpetuity. It is made available under aCC-BY 4.0 International license.

ESHGHI EPP Gating 1

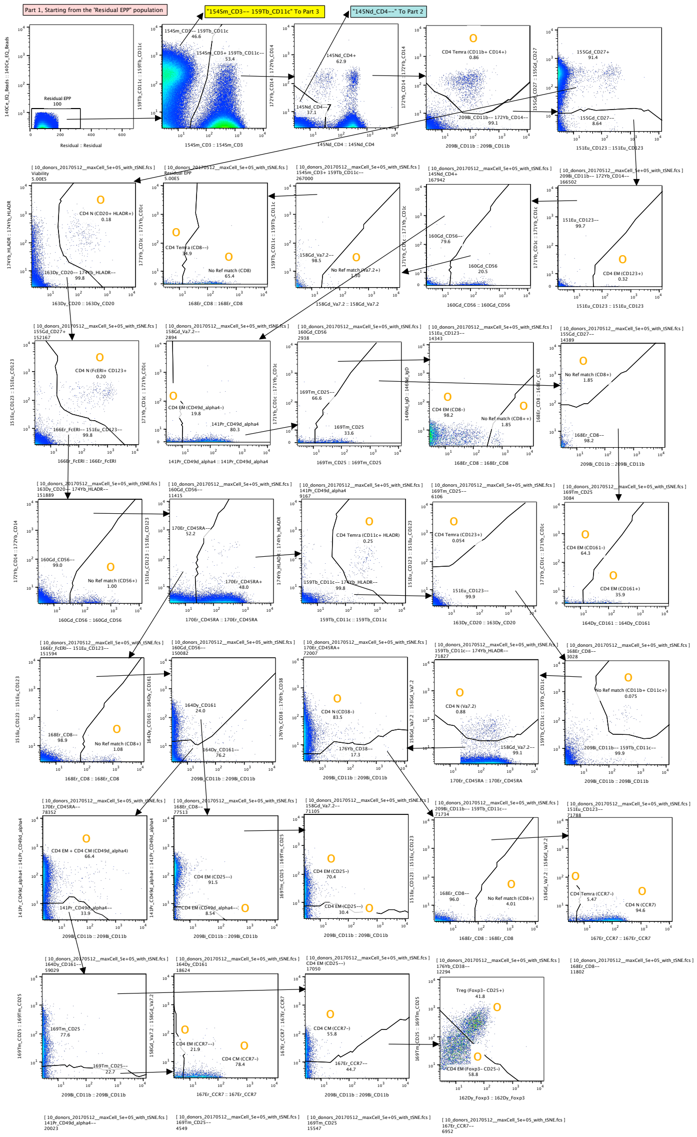

Figure 16: Eshghi, et al. EPP gating sequence starting from the "Residual" population in the reference gating in Eshghi, et al[6]. The EPP phenotypes (final leaf populations) are marked with O. This is the first of four parts needed to document the 105 final populations identified in the EPP analysis.

bioRxiv preprint doi: https://doi.org/10.1101/2024.11.20.624581; this version posted June 6, 2025. The copyright holder for this preprint (which was not certified by peer review) is the author/funder, who has granted bioRxiv a license to display the preprint in perpetuity. It is made available under aCC-BY 4.0 International license.

ESHGHI EPP Gating 2

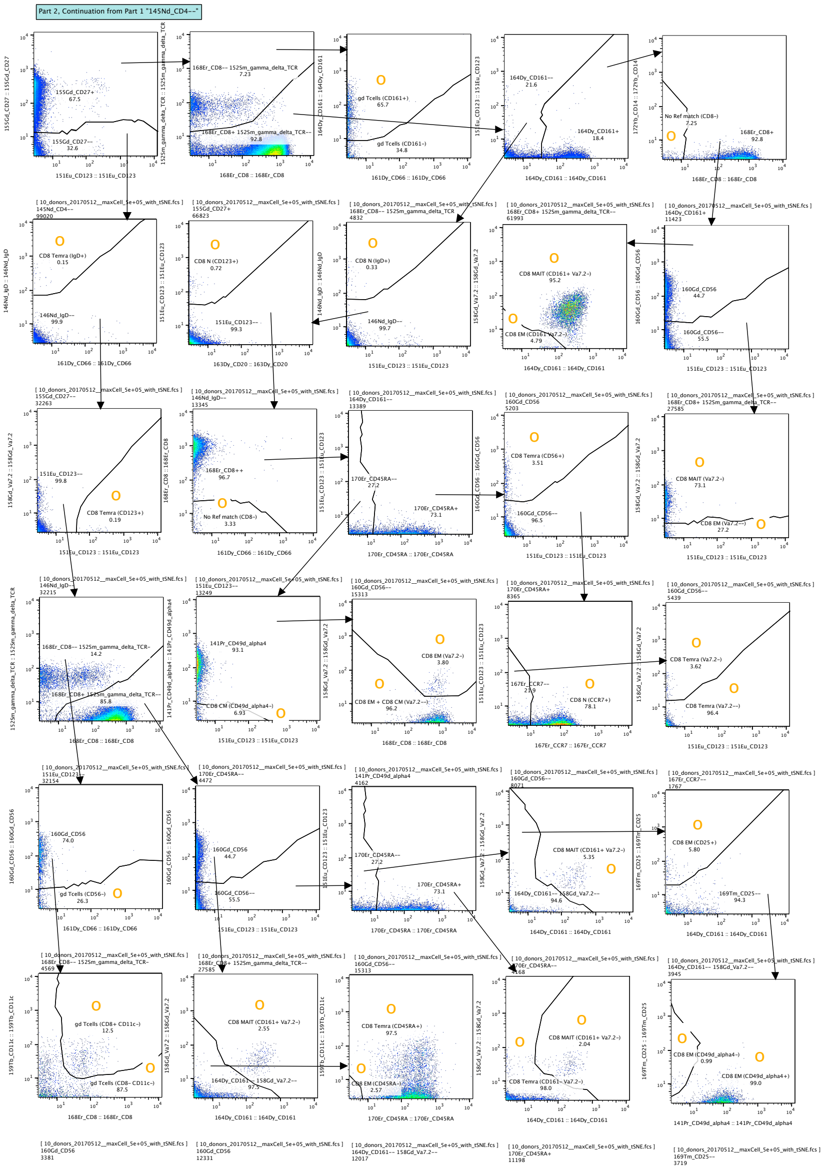

Figure 17: Part 2 of the EPP gating sequence for the Eshghi, et al[6], data set starting from the "145Nd_CD4–" population in Row 1 Column 3 of Part 1. The EPP phenotypes (final leaf populations) are marked with O.

bioRxiv preprint doi: https://doi.org/10.1101/2024.11.20.624581; this version posted June 6, 2025. The copyright holder for this preprint (which was not certified by peer review) is the author/funder, who has granted bioRxiv a license to display the preprint in perpetuity. It is made available under aCC-BY 4.0 International license.

ESHGHI EPP Gating 3

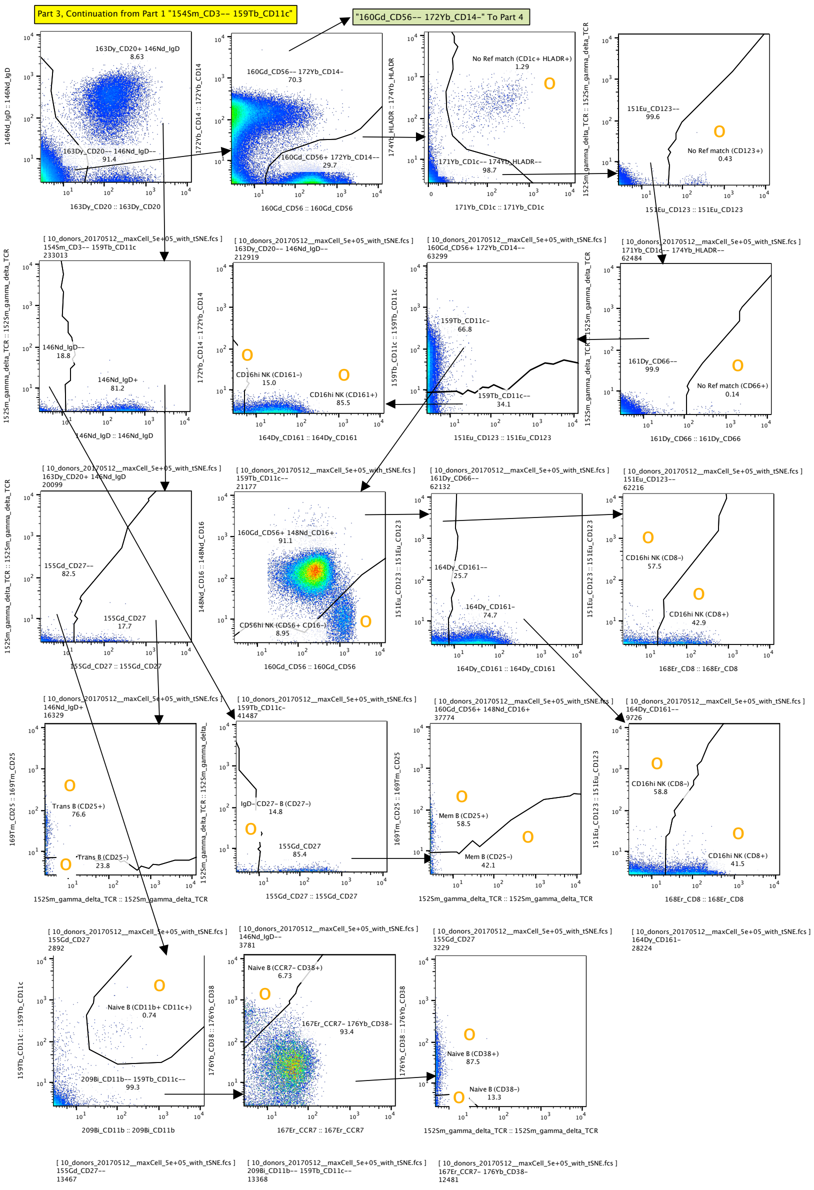

Figure 18: Eshghi, et al. Part 3 of the EPP gating sequence for the Eshghi, et al[6], data set starting from the "154Sm_CD3– 159Tb_CD11c" population in Row 1 Column 2 of Part 1. The EPP phenotypes (final leaf populations) are marked with O.

bioRxiv preprint doi: https://doi.org/10.1101/2024.11.20.624581; this version posted June 6, 2025. The copyright holder for this preprint (which was not certified by peer review) is the author/funder, who has granted bioRxiv a license to display the preprint in perpetuity. It is made available under aCC-BY 4.0 International license.

ESHGHI EPP Gating 4

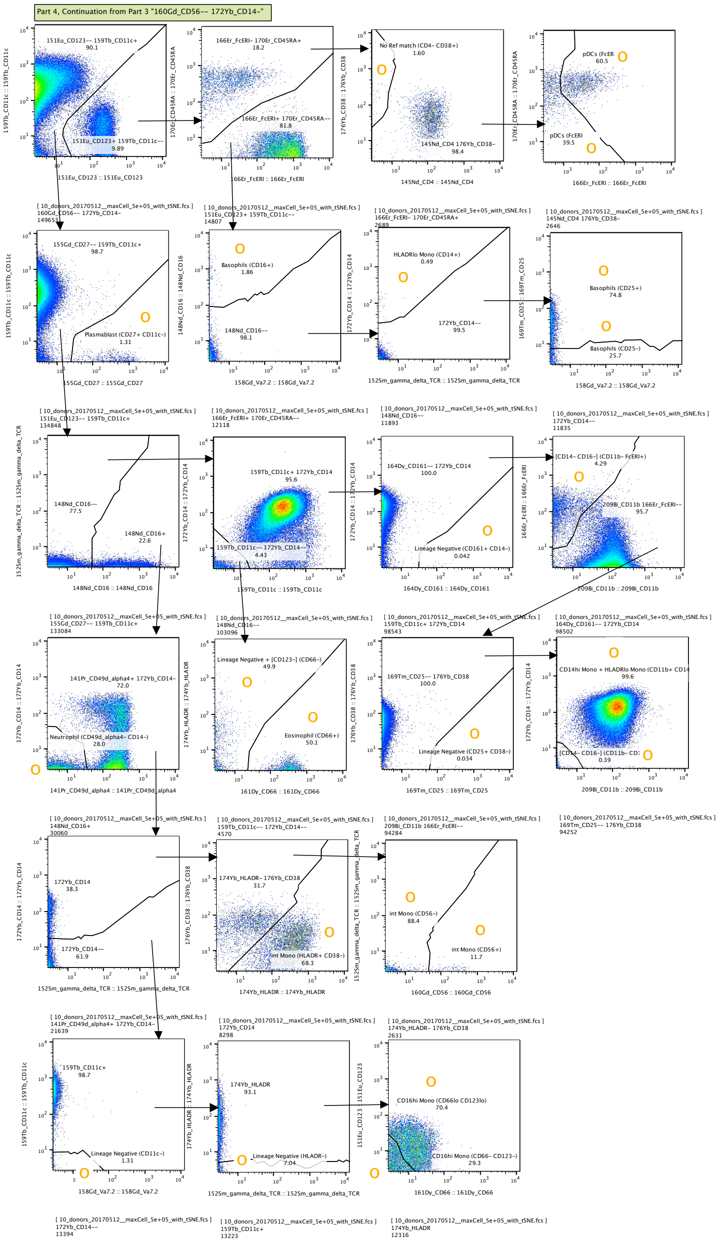

Figure 19: Part 4 of the EPP gating sequence for the Eshghi, et al[6], data set starting from the "160Gd_CD56– 172Yb_CD14-" population in Row 1 Column 2 of Part 3. The EPP phenotypes (final leaf populations) are marked with **O**.

bioRxiv preprint doi: https://doi.org/10.1101/2024.11.20.624581; this version posted June 6, 2025. The copyright holder for this preprint (which was not certified by peer review) is the author/funder, who has granted bioRxiv a license to display the preprint in perpetuity. It is made available under aCC-BY 4.0 International license.

ESHGHI Match Table-Full

Reference populations / EPP populations	events	1 CD14hi Mono	2 HLADRlo Mono	3 CD4 N	4 CD16hi NK	5 CD4 EM	6 CD4 CM	7 CD8 Temra	8 CD8 N	9 CD8 EM	10 Naive B	11 Basophils	12 CD16hi Mono	13 CD8 MAIT	14 gd Tcells	15 Neutrophil	16 int Mono	17 CD56hi NK	18 Mem B	19 Trans B	20 pDCs	21 CD4 Temra	22 Eosinophil	23 Treg	24 Lineage Negative	25 mDCs	26 CD8 CM	27 Plasmablast	28 [CD123-]	29 IgD- CD27- B	30 [CD1c- HLADR-]	Not matched
	events	80,750	11,310	70,143	56,449	45,354	29,702	29,765	23,383	16,756	13,016	11,906	11,776	8,886	9,049	7,929	8,126	3,298	3,041	3,179	2,519	8,120	2,409	4,833	2,729	1,833	2,266	1,350	782	858	338	
1 HLADRlo Mono + CD14hi Mono(CD11b CD14+)	94,536	79,523	10,810			3					3	19					714		10			3	95		70	11		1	27	97	162	2,988
2 CD14hi Mono(CD14+)	58	42	1									10					1													1		3
3 CD4 N(CD38-)	58,567			55,148											59							1,819	16	328	4							1,193
4 CD4 N(CCR7)	11,012			9,148											18	1						1,395	2	154	3							291
5 CD4 Temra(CD11b CD14)	1,444			535		94	38	41	52	12				16	25							241		38								352
6 CD4 N(Va7.2)	629			529																		19		4								77
7 CD4 N(CD20+ HLADR+)	278			209			1															24		17								27
8 CD4 N(CD123+ FcERI+)	297			145		45	59					2										18		17								11
9 CD4 N(CD11c+ HLADR)	179			102											1							47		7								22
10 CD4 N(CD123+)	38			18																	2	10		2								6
11 CD16hi NK(CD161-)	18,267				17,430						1							139		1										11		685
12 CD16hi NK(CD8-)	16,246				15,794													33												6		413
13 CD16hi NK(CD8)	11,569				11,328						1							9		1								1		4		225
14 CD16hi NK(CD8-)	5,374				5,146													32												3		193
15 CD16hi NK(CD8)	4,058				3,912													29												1		116
16 CD16hi NK(CD161-)	2,924				2,584													51										1		1		287
17 CD4 EM + CD4 CM(CD49d_alpha4)	39,316			2,740		17,755	14,985								24	5						1,073	15	1,371	29							1,319
18 CD4 EM(CD25-)	11,757			190		7,404	3,372								8							279	3	190	4							307
19 CD4 EM(CD8-)	5,934			3		4,124	25				1				5							1,068	3		47				3			655
20 CD4 EM(CD25- Foxp3-)	4,040					3,742									1							106			16							175
21 CD4 EM(CD25-)	5,124			92		2,995	1,806								12	1						101	1		2							114
22 CD4 EM(CD49d_alpha4-)	2,263			1		1,725	116									1						81		44	46				5			244
23 CD4 EM(CD161-)	1,935			4		1,556	93															125		51	4				3			99
24 CD4 EM(CD161)	1,088					978	17															39		19	1							34
25 CD4 EM(CCR7-)	956					904									1							21			3				1			26
26 CD4 EM(CD49d_alpha4-)	1,567			66		782	569															23		73	1							53
27 CD4 EM(CD8-)	1,028			1	1	594									1							395	1	1								34
28 CD4 EM(CD123+)	47			1		15	5								1						3	9	1									12
29 CD8 Temra(CD45RA+)	11,637				8			10,572	3	609				2	11			1					3				2					426
30 CD8 Temra(CD161- Va7.2-)	10,907				1			9,354	4	995				3	2								2		74		3					469
31 CD8 Temra(Va7.2-)	6,366							6,095							30										19							222
32 CD8 Temra(CD56+)	943				1			742	133						14												2					51
33 CD8 Temra(Va7.2-)	254							245																								9
34 CD8 Temra(CD38 Va7.2-)	90							37	33					13																		7
35 CD8 Temra(CD123+)	60							22	3	10		12			3												1					9
36 CD8 Temra(IgD+)	49							15	9		11				6														1			7
37 CD4 CM(CCR7-)	8,456			471		758	6,554															88		331	3							251
38 CD4 CM(CCR7-)	3,537			258		1,008	2,054								1							105			4							107
39 CD8 N(CCR7)	25,334							1,393	22,975						12	3			3	1			2		7		663					275
40 CD8 N(IgD+)	89							7	69											2												11
41 CD8 N(CD123+)	96							19	55	7																	5					10
42 CD8 EM + CD8 CM(Va7.2-)	14,277							570		10,123					5								3		17		1,277					2,282
43 CD8 EM(CD49d_alpha4)	3,666									2,796				4	3								1		31		3					828
44 CD8 CM(CD49d_alpha4-)	1,084							9		632															10		229					204
45 CD8 EM(Va7.2-)	1,424							502	22	629				16	6										2		28					219
46 CD8 EM(Va7.2-)	579							24		441																	37					77
47 CD8 EM(CD45RA-)	299									209																						90
48 CD8 EM(CD25)	226									151															5		15					55
49 Naive B(CD38-)	10,845				2						10,651		2							100	2								43			45
50 Naive B(CD38-)	1,596				1						1,368		1							183			1		3				18			21
51 Naive B(CCR7- CD38+)	893										847									2			1						12			31
52 Naive B(CD11b CD11c+)	100	3	1								83					2	1			2			1		1							6
53 Basophils(CD25-)	8,794		2									8,688							1		1		7		38					6	7	44
54 Basophils(CD25-)	3,051	3	1									2,952									5		1		34	2			1	3	4	45
55 Basophils(CD16+)	226		2									200	3				1								5							15
56 CD16hi Mono(CD66- CD123-)	8,662				3								8,365				52					1	1		20					13		207
57 CD16hi Mono(CD66- CD123-)	3,596		10		3			1			2		3,373				39					1			23					11		133
58 CD8 MAIT(CD38- Va7.2-)	5,061				1			101	25	99				4,472	8								1									354
59 CD8 MAIT(Va7.2-)	3,978							2		14				3,676	1								1									284
60 CD8 MAIT(CD161+ Va7.2-)	311							1		2				293																		15
61 CD8 MAIT(CD161+ Va7.2-)	225							8						200																		17
62 CD8 MAIT(CD161+ Va7.2-)	223									7				191	1																	24
63 gd Tcells(CD161)	3,152														3,135																	17
64 gd Tcells(CD8- CD11c-)	2,954				5			4							2,554	1																390
65 gd Tcells(CD161-)	1,689														1,655																	34
66 gd Tcells(CD56-)	1,178														992	1									13				2			170
67 gd Tcells(CD8 CD11c-)	419							1							406																	12
68 Neutrophil(CD14- CD49d_alpha4-)	8,420		32		24								6			7,880							13		112							353
69 int Mono(CD38- HLADR+)	5,623		4		1												5,458		1			1								9		149
70 int Mono(CD56-)	2,320		358								2					12	1,666			1									1	3		277
71 int Mono(CD56+)	303		37		6												188													1		71
72 CD56hi NK(CD16- CD56+)	3,677									1								2,972	2	1			2					1		2		696
73 Mem B(CD25-)	1,868										2								1,658	53								30	7	53		65
74 Mem B(CD25-)	1,353				1						1								1,185	14			1			1		54	4	42		50
75 Trans B(CD25-)	2,199																			2,131					1			7	13			47
76 Trans B(CD25-)	687																			662					1			4				20
77 pDCs(FcERI-)	1,592	3										7					1				1,543		1		3					2	1	31
78 pDCs(FcERI-)	1,054	3	2										8						1		954		1		34				1	4	5	41
79 CD4 Temra(CCR7-)	618														7							538	1	43								29
80 Eosinophil(CD66+)	2,287																			1			2,176		2							108
81 Treg(CD25+ Foxp3)	2,831					439																18		2,103	6				2			263
82 Lineage Negative + [CD123-](CD66-)	2,291		21								15	4							41	5			1		1,088				501	62		553
83 Lineage Negative(HLADR-)	901		26		31								18										3		610					1		212
84 Lineage Negative(CD11c-)	165				23														1				6		95				13			27
85 Lineage Negative(CD14- CD161)	41		1																						28						4	8
86 Lineage Negative(CD25+ CD38--)	30	1	1														1		2						21					1		3
87 mDCs(FcERI)	3,422	1,154			1	1											1		2			2			1	1,766		1		5		488
88 mDCs(FcERI-)	104	15															2		1	1					1	47				5		32
89 Plasmablast(CD11c- CD27+)	1,749		1																117	16	2				145			1,242	113			113
90 IgD- CD27- B(CD27-)	560	3			4						28	2				1	1						10		1				6	500		4
91 [CD1c- HLADR-](CD11b-CD14-)	383											3							10	1			2		23	6		1		5	155	177
92 No Ref match(CD8)	1,923				2	316									2							198	2									1,403
93 No Ref match(CD8-)	1,613				2														5	1					15			7	1			1,582
94 No Ref match(CD56+)	1,522			482		116	8								38							271	1	39								567
95 No Ref match(CD8+)	846														1										1							844
96 No Ref match(CD8-)	820				1																				2							817
97 No Ref match(CD1c+ HLADR+)	805				20													15						1						1		768
98 No Ref match(CD8+)	489									1													1									487
99 No Ref match(CD123+)	266				113													17												2		134
100 No Ref match(CD8+)	114																															114
101 No Ref match(CD66+)	83															11							26									46
102 No Ref match(CD8+)	57																															57
103 No Ref match(CD11b+ CD11c+)	53															10						1										42
104 No Ref match(CD49d_alpha4-)	38									18																	1					19
105 No Ref match(CD4- CD38+)	36											7							1		7								4	3		14

Figure 20: Supplementary: ESHGHI Match Table between the full set of 30 reference populations and 105 EPP phenotypes. The Column headers show the manually gated Reference populations and their event numbers. The Rows show the EPP-identified populations with cell type names from their matched Reference populations and indicators of markers used in the EPP separation. The associated +/- levels are based on population positions in the EPP graphs. The main table cells show the number of shared events between the corresponding Reference and EPP populations. GREEN highlights indicate dominant matches. The ORANGE cells indicate a second Reference population that contains part of an EPP phenotype.
